# SARS-CoV-2 BA.2.86 is susceptible to the neutralizing antibody MO11 targeting subdomain 1 despite the E554K mutation near the epitope

**DOI:** 10.1128/jvi.01389-24

**Published:** 2025-01-22

**Authors:** Natsumi Hasegawa, Mitsuhiro Nishimura, Rei Takamiya, Hanako Ishimaru, Yasuko Mori

**Affiliations:** 1 Division of Clinical Virology, Center for Infectious Diseases, Kobe University Graduate School of Medicine 12885 , Kobe, Hyogo, Japan; St. Jude Children's Research Hospital, Memphis, Tennessee, USA

**Keywords:** SARS-CoV-2, neutralizing antibodies

## LETTER

**Fig 1:**
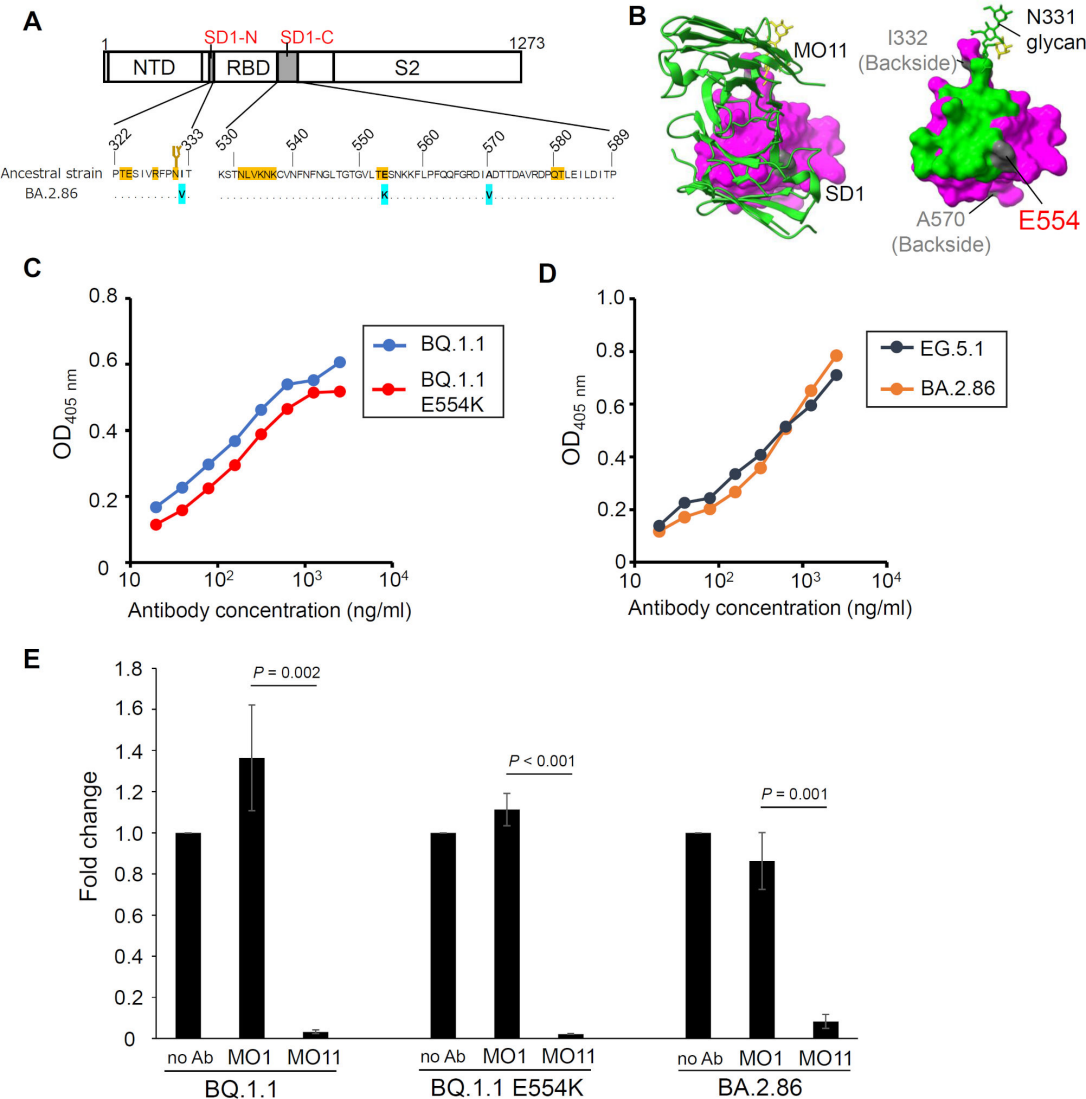
The SD1-targeting antibody MO11 still retains the neutralizing activity against SARS-CoV-2 BA.2.86. (**A**) The location of SD1 in spike (top) and the three mutations emerged in BA.2.86 (bottom) are shown. The residues in the footprint of the MO11 revealed by cryoEM analysis (1) are painted in orange. The N-glycosylation site at residue N331, which is also involved in the MO11 footprint, is indicated as a branch mark. (**B**) The binding mode (left) and footprint of MO11 on the SARS-CoV-2 spike SD1 (right, PDB: 8XI6) (1). The residues within 4 Å from MO11 are painted. The three mutation sites in the SD1, that is I332, A570, and E554, are painted in gray. (**C**) Reactivity of MO11 to BQ.1.1 spike ectodomain with or without E554K mutation. ELISA was performed as described previously (1). (**D**) Reactivity of MO11 to the EG.5.1 or BA.2.86 spike ectodomains. (**E**) Neutralizing activity of MO11 against SARS-CoV-2 spike pseudotyped VSV-ΔG (2, 3). The viruses were pseudotyped with spikes of BQ.1.1, BQ.1.1 with the E554K mutation (BQ.1.1 E554K), or BA.2.86 and mixed with indicated antibodies at a final concentration of 5 µg/mL at 37°C for 1 hour before infecting the VeroE6/TMPRSS2 cells. The luminescence signal was measured 1 day post-infection, and the values were normalized as the fold change relative to the values of no antibody (no Ab) condition. Means of the three independent experiments are shown with the standard deviations. The *P* values of Student’s *t*-test for the difference between the MO11 and the negative control (MO1) are shown above respective data.

The newly emerged SARS-CoV-2 variant BA.2.86 and its descendants, such as JN.1 and KP.3, have spread all over the world as of August 2024. These variants of the new generation share a spike mutation E554K on subdomain 1 (SD1) (Fig. 1A). Since the neutralizing antibody MO11, which we have reported in a previous article (1), targets SD1 by binding near the mutation site (Fig. 1B), the impact of the E554K mutation on MO11’s neutralizing activity needs to be addressed to evaluate the effectiveness.

The effect of the spike mutation E554K on the MO11’s binding ability was assessed by introducing the E554K mutation into the spike ectodomain of BQ.1.1, which is a BA.5 derivative prevalent in the first half of 2023 and has a characteristic R346T mutation in the RBD but no mutation in the SD1. In an ELISA experiment as in the previous study (1), the MO11 bound to the BQ.1.1 spike ectodomain in dose-dependent manners irrespective of the presence or absence of the E554K mutation (Fig. 1C). Interestingly, the MO11 was also found to retain the binding ability to the BA.2.86 spike ectodomain without any noticeable difference from that to the spike ectodomain of EG.5.1, which is a derivative of the XBB strain with no SD1 mutation (Fig. 1D). As the MO11 has neutralizing activity against the live virus of EG.5.1 with an IC_50_ of 0.21 µg/mL (1), the retained binding ability to the mutant spike implies retention of the neutralizing activity.

A neutralization assay system using the vesicular stomatitis virus (VSV)-based pseudotyped virus was employed (2, 3) to assess the neutralization activity. The spike protein harboring mutations of the BQ.1.1 strain is expressed on the cell surface of the HEK293T cells (2), and the VSV-ΔG, which contains the luciferase gene in replacement of the VSV G gene, is pseudotyped by the spike. MO11 could neutralize the BQ.1.1 spike pseudotyped virus (Fig. 1E, left), while our previous RBD-targeting antibodies, MO1 (4), could not neutralize the pseudovirus, as consistent with the results in live virus-neutralizing assays using BQ.1.1 (1, 4). BQ.1.1 spike-E554K-pseudotyped virus, prepared using BQ.1.1 spike with the E554K mutation for pseudotyping, was also neutralized by MO11 (Fig. 1E, middle). Finally, we tested the BA.2.86 spike-pseudotyped virus, and the MO11 was shown to retain neutralizing activity (Fig. 1E, right).

**Fig 2:**
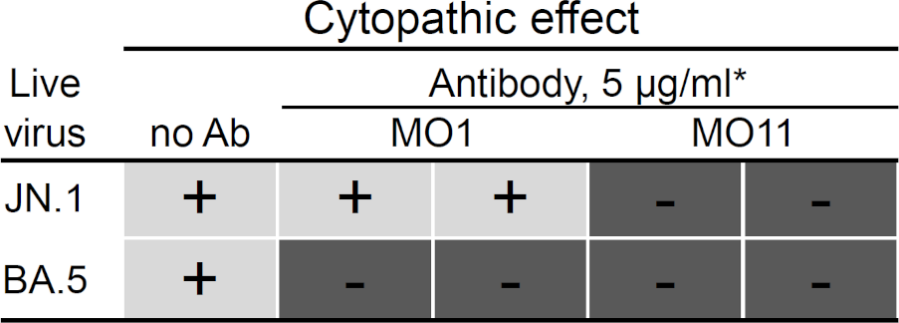
MO11 exhibited neutralizing activity against JN.1 in a live virus neutralizing assay. VeroE6/TMPRSS2 cells were infected with JN.1 (EPI_ISL_19175672) or BA.5 (EPI_13241867) live viruses around 100 median tissue culture infectious dose (TCID50) with the indicated antibodies at a final concentration of 5 µg/mL. The neutralizing activity is determined as follows: +, cytopathic effect is observed by 4 dpi; −, no cytopathic effect is observed by 4 dpi, indicating neutralization by the antibody. The same result is confirmed in two repeated experiments. “*” indicates that the two rows for each antibody represent the duplicate result in one experiment.

We also performed a live virus neutralization experiment using the JN.1 strain, which has spread worldwide in the first half of 2024. The spike of JN.1 is equivalent to that of BA.2.86 except for one mutation, L455S, in the RBD. MO11 showed neutralizing activity against the live virus of JN.1 as the cytopathic effect was not observed by adding MO11, while JN.1 was not neutralized by MO1 (Fig. 2). This result is consistent with the neutralization experiment using the BA.2.86 spike pseudotyped virus. All the data obtained here indicated the retained neutralizing activity of MO11 against the viruses of BA.2.86 lineage, which contain the E554K mutation.

The retained neutralizing activity indicates that a conserved neutralizing epitope is still available on the spike SD1. The E554K mutation is suggested to contribute to the explosive prevalence of the BA.2.86, indicating the selective pressure posed by SD1-targeting antibodies (5). The E554K has no definitive influence on MO11’s activity, and it is explained by the structural observation that the E554 is located around the rim of the footprint (Fig. 1B). As far as we know, MO11 is a unique SD1-targeting antibody effective against BA.2.86. All other SD1-targeting antibodies recognize E554 as a key residue in their epitopes and retain no neutralizing activities against BA.2.86 (5, 6). Thus, MO11 may have the potential for clinical application against BA.2.86 lineages, such as JN.1 and KP.3, which are circulating worldwide. If MO11-like SD1-targeting antibodies pose selection pressure on BA.2.86 and its descendants, novel mutations on SD1 may emerge in the future SARS-CoV-2 variants.

## References

[1] Ishimaru H, Nishimura M, Shigematsu H, Marini MI, Hasegawa N, Takamiya R, Iwata S, Mori Y. 2024. Epitopes of an antibody that neutralizes a wide range of SARS-CoV-2 variants in a conserved subdomain 1 of the spike protein. J Virol 98:e0041624. doi:10.1128/jvi.00416-24 38624232 PMC11092320

[2] Anzai I, Fujita J, Ono C, Kosaka Y, Miyamoto Y, Shichinohe S, Takada K, Torii S, Taguwa S, Suzuki K, Makino F, Kajita T, Inoue T, Namba K, Watanabe T, Matsuura Y. 2024. Characterization of a neutralizing antibody that recognizes a loop region adjacent to the receptor-binding interface of the SARS-CoV-2 spike receptor-binding domain. Microbiol Spectr 12:e0365523. doi:10.1128/spectrum.03655-23 38415660 PMC10986471

[3] Tani H, Kimura M, Tan L, Yoshida Y, Ozawa T, Kishi H, Fukushi S, Saijo M, Sano K, Suzuki T, Kawasuji H, Ueno A, Miyajima Y, Fukui Y, Sakamaki I, Yamamoto Y, Morinaga Y. 2021. Evaluation of SARS-CoV-2 neutralizing antibodies using a vesicular stomatitis virus possessing SARS-CoV-2 spike protein. Virol J 18:16. doi:10.1186/s12985-021-01490-7 33435994 PMC7801864

[4] Ishimaru H, Nishimura M, Tjan LH, Sutandhio S, Marini MI, Effendi GB, Shigematsu H, Kato K, Hasegawa N, Aoki K, Kurahashi Y, Furukawa K, Shinohara M, Nakamura T, Arii J, Nagano T, Nakamura S, Sano S, Iwata S, Okamura S, Mori Y. 2023. Identification and analysis of monoclonal antibodies with neutralizing activity against diverse SARS-CoV-2 variants. J Virol 97:e0028623. doi:10.1128/jvi.00286-23 37191569 PMC10308935

[5] Zhou D, Supasa P, Liu C, Dijokaite-Guraliuc A, Duyvesteyn HME, Selvaraj M, Mentzer AJ, Das R, Dejnirattisai W, Temperton N, Klenerman P, Dunachie SJ, Fry EE, Mongkolsapaya J, Ren J, Stuart DI, Screaton GR. 2024. The SARS-CoV-2 neutralizing antibody response to SD1 and its evasion by BA.2.86. Nat Commun 15:2734. doi:10.1038/s41467-024-46982-6 38548763 PMC10978878

[6] Wang Q, Guo Y, Liu L, Schwanz LT, Li Z, Nair MS, Ho J, Zhang RM, Iketani S, Yu J, Huang Y, Qu Y, Valdez R, Lauring AS, Huang Y, Gordon A, Wang HH, Liu L, Ho DD. 2023. Antigenicity and receptor affinity of SARS-CoV-2 BA.2.86 spike. Nature New Biol 624:639–644. doi:10.1038/s41586-023-06750-w 37871613

